# Similarity corpus on microbial transcriptional regulation

**DOI:** 10.1186/s13326-019-0200-x

**Published:** 2019-05-22

**Authors:** Oscar Lithgow-Serrano, Socorro Gama-Castro, Cecilia Ishida-Gutiérrez, Citlalli Mejía-Almonte, Víctor H. Tierrafría, Sara Martínez-Luna, Alberto Santos-Zavaleta, David Velázquez-Ramírez, Julio Collado-Vides

**Affiliations:** 1 Computational Genomics, Centro de Ciencias Genómicas, Universidad Nacional Autónoma de México (UNAM). A.P., 565-A Cuernavaca, Morelos, 62100 México; 2 Instituto de Investigaciones en Matemáticas Aplicadas y en Sistemas (IIMAS), Universidad Nacional Autónoma de México (UNAM), Mexico City, México; 3 Department of Biomedical Engineering, Boston University, Boston, Massachusetts, USA

**Keywords:** Corpus, Similarity, Transcriptional-regulation, Genomics

## Abstract

**Background:**

The ability to express the same meaning in different ways is a well-known property of natural language. This amazing property is the source of major difficulties in natural language processing. Given the constant increase in published literature, its curation and information extraction would strongly benefit from efficient automatic processes, for which corpora of sentences evaluated by experts are a valuable resource.

**Results:**

Given our interest in applying such approaches to the benefit of curation of the biomedical literature, specifically that about gene regulation in microbial organisms, we decided to build a corpus with graded textual similarity evaluated by curators and that was designed specifically oriented to our purposes. Based on the predefined statistical power of future analyses, we defined features of the design, including sampling, selection criteria, balance, and size, among others. A non-fully crossed study design was applied. Each pair of sentences was evaluated by 3 annotators from a total of 7; the scale used in the semantic similarity assessment task within the Semantic Evaluation workshop (SEMEVAL) was adapted to our goals in four successive iterative sessions with clear improvements in the agreed guidelines and interrater reliability results. Alternatives for such a corpus evaluation have been widely discussed.

**Conclusions:**

To the best of our knowledge, this is the first similarity corpus—a dataset of pairs of sentences for which human experts rate the semantic similarity of each pair—in this domain of knowledge. We have initiated its incorporation in our research towards high-throughput curation strategies based on natural language processing.

## Background

Expressing the same approximate meaning with different wording is a phenomenon widely present in the everyday use of natural language. It shows the richness and polymorphic power of natural language, but it also exhibits the complexity implied in understanding the conveyed meaning. Due to these characteristics, paraphrase identification is necessary for many Natural Language Processing (NLP) tasks, such as information retrieval, machine translation, and plagiarism detection, among others. Although strictly a “paraphrasis” refers to a rewording that states the same meaning, i.e., its evaluation should only result in true or false, frequently a graded paraphrasing is needed. This graded paraphrasing is often called Semantic Textual Similarity (STS).

Textual similarity depends on particular text features, domain relations, and the applied perspective; therefore, textual similarity has to be defined according to the context. This context specification presupposes the delineation of the *kind of textual similarity* desired, e.g., assigning grades of importance to the syntactic parallelism, to the ontological closeness, to the statistical representations likeness, etc.

It is not a simple endeavor to explicitly state these grades of importance. The difficulty stems from the fact that it is very complicated to envisage all possible language feature variations to express the same idea, and so to have a broad perspective and to identify which features or relations are important. It is for these steps that a paraphrase corpus is a very useful instrument, because it implicitly captures those nuances.

There are several paraphrase corpora available, both for general and specific domains. However, as stated before, these corpora are very sensitive to the aimed task and to the targeted domain. Hence, when a task or domain is very specific and the available corpora do not fit, an ad hoc corpus has to be built. This is the case for the biomedical curation of the literature about the regulation of transcription initiation in bacteria, a specific domain of knowledge within the biomedical literature.

RegulonDB^1^ [1] is a manually curated standard resource, an organized and computable database, about the regulation of gene expression in the model enterobacteria *Escherichia coli* K-12. It aims at integrating within a single repository all the scattered information in the literature about genetic regulation in this microorganism, including elements about transcriptional regulation, such as promoters, transcription units (TUs), transcription factors (TFs), effectors that affect TFs, active and inactive conformations of TFs, TF binding sites (TFBSs), regulatory interactions (RIs) of TFs with their target genes/TUs, terminators, riboswitches, small RNAs, and their target genes. We are capable of keeping up to date with the literature thanks to constant manual curation in an effort initiated close to 20 years ago. However, the pace of curation tends to lag behind the number of publications, motivating the implementation of automatic curation processes^2^. Certainly, biocuration typically accelerates with the emergence of novel technologies, and furthermore, we believe that the depth and detail of the description of what is extracted from the literature could be increased significantly. As shown in the most recent publication of RegulonDB [2], the number of curated objects has increased over the years. Finally, another major motivation stems from the fact that microbial genomes have been constructed under similar evolutionary principles as *E. coli*; thus, the methods that can be trained with literature for *E. coli* should be very well applicable to the literature on gene regulation in other microbial organisms, for which the literature has not been subject to curation. RegulonDB plays an important role in scientific research: it has been cited in more than 1700 scientific publications.

As an ongoing effort to enrich the already curated information and to improve the curation process, we are developing NLP tools, some of which rely on STS. The goal with these STS assessment tools is to discover statements, in different publications, connected by their meaning. One of the direct contributions to the curation process could be to facilitate the discovery of supporting evidence for a piece of curated information. Table 1 shows a pair of sentences, from different publications, that express very similar meanings and that provide supporting evidence for each other. These pairs of sentences exemplify what is intended to be annotated within our corpus and, thus, the kind of annotations that we expect to produce through machine learning models trained with this corpus. Due to the very specific nature of our domain, we built the ad hoc graded paraphrase corpus to be used as a training and evaluation source of truth for our NLP tools.

**Table 1:** Examples of sentences of different publications that express very similar meanings

Sentence	Publication title
There is, however, some evidence that increased rob expression occurs in glucose—and phosphate—limited media in the stationary phase of cell growth, attributable to activation by factor rpoS.	MarA-mediated transcriptional repression of the rob promoter. (PMID: 16478729)
A similar rpoS dependency was observed for glucose-limited or phosphate-limited growth in which rob::lacZ transcription increased 5-fold.	Posttranscriptional activation of the transcriptional activator Rob by dipyridyl in Escherichia coli. (PMID: 11844771)

In the following sections, we first describe the methodology followed to build our corpus, then we analyze it quantitatively, and finally we briefly mention the immediate foreseen uses of the corpus.

### Related work and motivation

STS aims to measure the degree of semantic equivalence between two fragments of text. To achieve this, it tries to unveil the meaning conveyed by a textual expression and compare it with the meaning conveyed by another one. The comparison’s result is a graded similarity score that ranges from an exact semantic match to a completely independent meaning, passing through a continuous scale of graded semantic parallelism. This scale intuitively captures the notion that a pair of texts can share different aspects of meaning at different levels [3], i.e., they could differ in just some minor details, they could share a common topic and important details, or they could share only the domain and context, etc. Another characteristic of STS is that it treats similarities between two texts as bijective, setting this task apart from textual entailment, where the relation is directed and cannot be assumed true in the inverse direction.

Many NLP tasks, such as machine translation, question answering, summarization, and information extraction, potentially benefit from this quantifiable graded bidirectional notion of textual similarity. Building this kind of corpus is difficult and is labor-intensive, and that is why there are not as many corpora of this kind as might be expected, given their usefulness.

In recent years, the most notorious efforts on the STS task and their corresponding corpus constructions were tackled by the *Semantic Evaluation Workshop* (SEMEVAL) [3]. The *SEMEVAL* corpus consists of 15,000 sentence pairs from different sources, with the *Microsoft Research Paraphrase* (MSRP) and PASCAL VOC [4] corpora among them. The SEMEVAL corpus was annotated through crowdsourcing, using a scale from 5 (identical) to 0 (completely unrelated).

Another corpus that is useful for STS is the *User Language Paraphrase* corpus (ULPC) [5]. This corpus was built by asking students to rephrase target sentences. As a result, 1998 sentence pairs were annotated with ratings ranging from 1 to 6 for 10 paraphrasing dimensions; entailment and lexical, syntactic, and semantic similarities were among those dimensions.

The *SIMILAR* corpus [6] is the product of a qualitative assessment of 700 pairs of sentences from the *MSRP* corpus; in addition to providing word-to-word semantic similarity annotations, it also supplies a qualitative similarity relationship—identical, related, context, close, world knowledge, or none—between each pair of sentences.

Among corpora that do not rely on graded similarity but instead on binary paraphrases, there are important corpora, such as the *MSRP* corpus [7]. It is one of the first major public paraphrase corpora, comprising 5801 new sentence pairs, of which 67% were judged “semantically equivalent” by two human judges. In the Q&A field, another corpus, *The Question Paraphrase* corpus [8], was built by collecting from *WikiAnswers* 7434 sentences formed by 1000 different questions and their paraphrases.

All these corpora target general domains and were sourced mainly from the news, making it very difficult to fit them into a specific topic such as ours: *bacterial transcriptional regulation*. Closer to our domain is the BIOSSES corpus [9]. It is formed by 100 pairs of sentences from the biomedical domain which were rated following the guidelines of the STS SEMEVAL task. The candidate sentences were collected from the set of articles that cited at least 1 of 20 reference articles (between 12 and 20 citing articles for each reference article). Those sentence pairs that cited the same reference article were selected. Articles were taken from the *Biomedical Summarization Track Training Dataset* from the *Text Analysis Conference*.

Due to the extension of the biomedical domain and the small size of the BIOSSES corpus, most likely it does not capture the nuances of our subject of study. For this reason, we decided to build our own corpus of naturally occurring non-handcrafted sentence pairs within the subject of *regulation of gene expression* in *E. coli* K-12. The semantic similarity grade of each pair was evaluated by human experts of this field.

## Methods

### Corpus design

A corpus is “a collection of pieces of language text in electronic form, selected according to external criteria to represent, as far as possible, a language or language variety as a source of data for linguistic research” [10]. Before building a corpus, the textual source set, the evaluation rules, the corpus size, and other characteristics must be defined. This design should be, as much as possible, informed and principled so that the resulting corpus fulfills the desired goals. The decisions involved within the axes of consideration [10] for the corpus construction are the following.

The *sampling* policy defines where and how the candidate texts are going to be selected, following three main criteria: the *orientation*, in this case a contrastive corpus with the aim of showing the language varieties that express the same meaning (semantic similarity); the *selection criteria* that circumscribe candidates to written sentences (origin and granularity) in English (language) taken from scientific articles (type) on the topic of genetic regulation (domain), where the sentence attitude^3^ is irrelevant and a specific content is not required; finally, the *sampling* criteria consists of preselection of sentence pairs through a very basic STS component followed by a filtering process to keep the same number of exemplars for each similarity grade, i.e., a balanced candidate set.

The corpus *representativeness* and *balance* refer to the kind of features and to the distribution of those features in the exemplars; hence, these characteristics determined the usage possibilities of the corpus. In this sense, sentences containing any biological element or knowledge were preferred. It was more important that all similarity grades were represented within the corpus and preferably in equal proportions. Our main analysis axis was the semantic similarity between pairs of sentences and not the topic represented by each sentence, the sentences’ specialization or technical level, nor the ontological specificity of the terms in the sentence.

The orientation of a corpus’ *topics* impacts directly the variety and size of the resulting vocabulary. Whereas embracing more topics can broaden the possibilities for use of the corpus, this can also have negative consequences in the semantic similarity measures due to the increased chances of the same term having different meanings for different topics (ambiguity). Consequently, a limited set of topics was preferred. We intended for the corpus to be representative of the genetic regulation literature. It is worth noting that it was not limited to those sentences specifically about genetic regulation but all kinds of sentences present in the corresponding literature. The corpus *homogeneity* was tackled by stripping out those sentences considered too short (less than 10 words) [11] and those sentences that were not part of the main body of the article^4^.

Finally, a corpus’ *size* should be dependent on the questions that it is aimed to answer and the type of tasks where it can be applied [12, 13]. However, in practice it is largely restrained according to available resources (time, money, and people). Our main goals are to train our STS system and to measure its performance. Because our STS system is based on the combination of several similarity methods, it is difficult to estimate the required number of cases that would make it a significant training source, because this varies for each type of metric. For example, one of the most demanding methods on training data is neural networks, whose complexity can be expressed based on the number of parameters (*P*), and it is common practice to have at least *P*^2^ training cases. This would result in thousands of training cases, which is out of our reach. Thus, we focused on the second goal, to measure the STS system performance. We planned to measure the Pearson’s correlation between the computed system similarity and that generated by human experts (corpus). According to [14], considering a medium-size effect (r = 0.30), a significance level of 0.05, and a power of 80%, 85 samples would be enough. However, [15] and [16] suggested a minimum sample size of 120 cases in order to allow not only a Pearson’s correlation analysis but also a regression analysis. With this in mind, we decided to generate a corpus of 170 sentence pairs, i.e., a number of pairs just above those thresholds.

Lastly, as a validity exercise, we compared our design decisions versus those taken in other corpora, for example, the MSRP corpus. In the construction of the MSRP corpus [7], several constraints were applied to narrow the space of possible paraphrases. However, in our opinion and for our specific purpose, these guidelines limit the aspects of semantic similarity that the corpus could capture. For example, only those pairs of sentences with at least 3 words in common and within a range of Levenshtein edit distance were considered, but these parameters constrain similarity, at least to a certain extent, to a textual one; it was required that for a pair to be a candidate, the length in words of the shorter sentence be more than 66% of the length of the longer sentence, thus limiting the possibility for the corpus to represent cross-level semantic similarity [17], a phenomenon of sentences with different lengths. It is also noteworthy that the MSRP corpus has an agreed consensus that 67% of the proposed sentence pairs are paraphrases, meaning that the majority of sentences are semantically equivalent and, therefore, other grades of similarity and even nonsimilarity are underrepresented.

#### Compiling the corpus

As stated in the sampling criteria of the corpus design, the selection of candidate pairs was performed using a basic STS process that automatically assigned continuous similarity scores between 0 and 1 inclusive, where 1 represented exact semantic equivalence^5^ and 0 indicated a totally unrelated meaning. The referred *basic STS process* was performed by a tool that we developed to compare the semantic similarity of two sentences using only their word embeddings. The strategy consisted of averaging the embeddings of the sentence words to produce a sentence embedding and compute the cosine between both sentence embeddings as a measure of their similarity. This strategy is well known as a good baseline for this kind of task. It is worth noting that the embeddings were trained on RegulonDB’s literature—Transcriptional Regulation domain (further details of this strategy and the word embedding training are presented in [18]). Next, the final candidate sentences were selected by a balanced stratified random sampling from those prerated sentence pairs.

This process was applied to two different sets: the *anaerobiosis FNR* (Fumarate and Nitrate Reductase regulatory protein) subset formed by articles about anaerobiosis; and the *general* set, consisting of sentences taken by randomly sampling of all of RegulonDB’s articles (5963 publications). The former subset was manually built by an expert curator who selected, from anaerobiosis articles, sentences that she considered relevant within the subject. To generate the latter subset, we first extracted the textual content (sentences) from the 5963 publications (PDFs) found in the literature of RegulonDB by using a tool that we built for this purpose. Then, as a naive approach to only focus on sentences belonging to the article’s main sections (e.g., methods, results, discussion), we discarded the first 30% and the last 30% of sentences from each article. Finally, we randomly chose two sentences from each article.

**Fig. 1:**
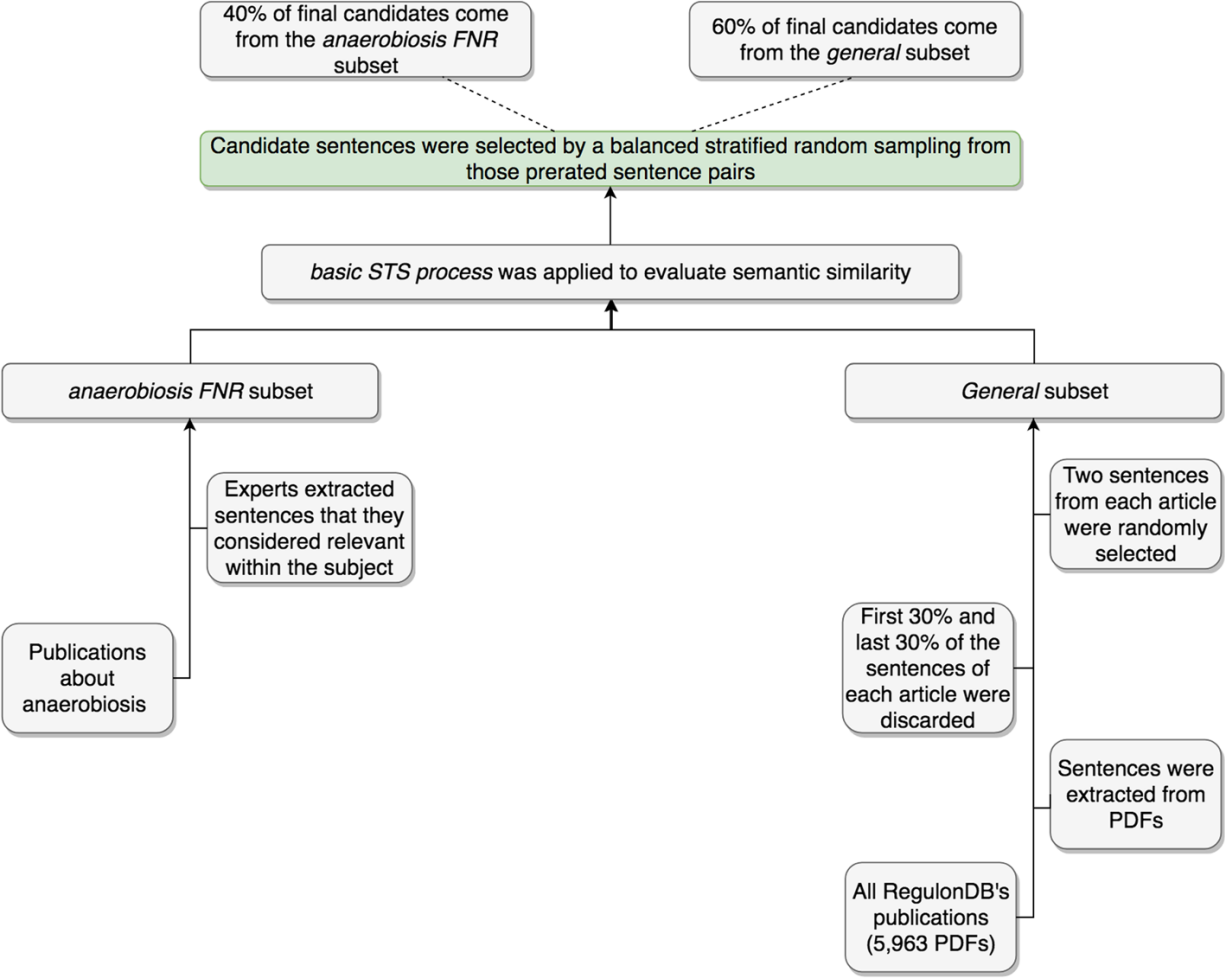
Corpus compilation pipeline. This pipeline, from bottom to top, shows the steps that were taken to compile the corpus (171 sentence pairs) that was later evaluated by annotators regarding the semantic similarity between the sentence pairs. First, two subsets, the anaerobiosis-FNR and the more general one, were compiled using different strategies. Then, a basic STS process was applied to both subsets in order to have a preliminary semantic similarity evaluation. This preliminary evaluation was used to select candidate sentences, creating a corpus that ended up with 40% of sentences from the anaerobiosis subset and 60% from the general subset

The resulting corpus is formed by pairs of sentences, of which 40% come from the anaerobiosis FNR subset and 60% from the general subset. A big picture of the described pipeline is shown in Fig. 1.

### Annotation design

In addition to the corpus design, it was necessary to delineate the semantic similarity rating process. We followed a similar rating scale to the one used in SEMEVAL. This is an ordinal scale ranging from 0 to 4, where a *Sentence Pair Similarity Score* (SPSS) of 0 represents a totally disconnected semantic relation between two sentences and 4 conveys an exact semantic match, with the three middle scores indicating similarity shades, as shown in Table 2.

**Table 2:** Rating scale

SPSS	Description
4	The two sentences are completely or mostly equivalent, as they mean the same thing.
3	The two sentences are roughly equivalent, but some important information differs/is missing.
2	The two sentences are not equivalent but share some details.
1	The two sentences are not equivalent but are on the same topic.
0	The two sentences are on different topics.

Seven human experts, who are coauthors of the present article, comprised the set of annotators for the task. We decided to apply a *non-fully crossed study design*^6^ in which different sentence pairs were rated by different subsets of 3 annotators, i.e., each sentence pair would be rated by 3 annotators selected by chance from the set of the 7 human experts. Some studies have shown that 2 evaluations per item can be enough [19], but we considered that 3 annotators per item would allow evaluation of a larger number of exemplars, and also that 3 is the smallest number to provide a median when there is no consensus and a discrete final score is desired.

Due to the fact that “what is considered semantically equivalent” is prone to be biased by personal subjective considerations, it was necessary to homogenize the annotation process among raters. This was done by a training period of 4 iterative sessions to help annotators become familiar with the annotation guidelines and the corpora to be rated, and also to refine annotation guidelines. During this training, each session consisted of evaluating a small subset of sentence pairs, and at the end of each session, disagreements were discussed and solved and annotation guidelines were more precisely defined. This training period was considered concluded when a minimum annotator interagreement was achieved or the annotators considered that they fully understood the annotation guidelines.

#### General guidelines

In order to make the annotation process less subjective, some general guidelines were initially given to raters. These were collected from other corpus-building experiments [20] and from our own observations, including:

- *Order*. Clauses in a compound sentence can be arranged in a different order without implying a change in its meaning.
- *Missing clauses*. In complex or compound sentences, if a clause is present in one and missing in the other, it does not automatically result in a zero similarity. It depends on the grade of importance of the shared information.
- *Adjectives*. Missing adjectives in principle do not affect similarity.
- *Enumerations*. Missing elements can produce a minor decrease in the similarity score unless enumeration conveys the main sentence meaning. Reordering is considered equivalent.
- *Abbreviations*. Abbreviations are considered equivalent, e.g. “vs” and “versus.”
- *Hypernyms and hyponyms*. The two forms share a grade of similarity, e.g., “sugar substance” vs “honey” vs “bee honey.”
- *Compound words*. Some terms are semantically equivalent to multiterm expressions, e.g., “anaerobiosis” and “in the absence of oxygen,” “oxidative and nitrosative stress transcriptional regulator” and “oxyR,” or “hemorrhage” and “blood loss.”
- *Generalization or abstractions*. Consider that two textual expressions share some grade of semantic similarity if one is a generalization or abstraction of the other, e.g., 8 vs “one-digit number.”

#### Consensual refinement

General guidelines were subsequently refined and enriched during the consensus sessions.

As a first approximation to clarify the rating scale in our context, it was decided we would use the class of RegulonDB objects as topic markers within the sentences. RegulonDB contains objects of the following classes: Gene, Gene Product, Protein, Motif, Promoter, Transcription Unit (TU), Regulatory Interaction (RI), Reaction, Transcription Factor (TF), and Growth Condition (GC). Next, we provide example cases for each score that help to clarify our similarity scale.

##### SPSS of 4.

Both sentences have in common the same objects and express the same meaning. i.e., they are paraphrases of each other. The following pair of sentences serve to illustrate this grade:

- *This would mean that the IS5 element is able to provide FNR regulatory sites if inserted at appropriate positions.*
- *In any case, insertion of an IS5 element is able to increase FNR-dependent expression or to place genes under FNR control.*

##### SPSS of 3.

Both sentences share the same objects and other elements of their meaning. However, one of the sentences lacks relevant elements, does not refer to the same objects, or arrives at different conclusions. Some cases we could envision are that both sentences refer to the same Gene and share all other information, except that in one the gene is activated and in the other it is repressed; sentences referencing the same RI but that differ in terms of the RI’s conditions; both sentences almost paraphrase each other, but one has more details.

The relation between the next pair of sentences exemplifies the last case:

- *These results confirm that the N-terminal domain of NikR is responsible for DNA recognition.*
- *In preliminary experiments, we have also found that a subset of mutations within the DNA region protected by the N-terminal domain reduce the affinity of NikR for the operator—data not shown.*

##### SPSS of 2.

Both sentences share at least one specific object and some other similarities, for example, a pair of sentences that refer to the same TF (see example (a)). An interesting singularity from the expert evaluation was the observation that “aerobic” and “anaerobic” conditions are related, since they both refer to oxygen availability. Therefore, in this corpus, contrasting conditions like these have a certain degree of similarity (see examples (a) and (b)).

- Example (a)
  - *The fnr mutant was thus deficient in the anaerobic induction of fumarate reductase expression.*
  - *Aerobic regulation of the sucABCD genes of Escherichia coli, which encode K-ketoglutarate dehydrogenase and succinyl coenzyme A synthetase: roles of ArcA, Fnr, and the upstream sdhCDAB promoter.*
- Example (b)
  - *Aerobic regulation of the sucABCD genes of Escherichia coli, which encode K-ketoglutarate dehydrogenase and succinyl coenzyme A synthetase: roles of ArcA, Fnr, and the upstream sdhCDAB promoter.*
  - *Transcription of the fdnGHI and narGHJI operons is induced during anaerobic-growth in the presence of nitrate.*

##### SPSS of 1.

Both sentences have the same object class in common, but the specific object is different. Since Gene and GC objects are highly common in RegulonDB’s literature, it was decided that sharing only these classes is not a sufficient condition for sentences to be rated with an SPSS of 1. When comparing a sentence that mentions a TF with another one that mentions any other object (or GC) that refers to the same process in which the TF is involved, an SPSS of 1 has to be assigned to the sentence pair. An SPSS of 1 was also considered in cases when both sentences referred to sequences and genes, even when neither the sequences nor the mentioned genes were the same. The following pair of sentences is an example of this grade:

- *The fnr mutant was thus deficient in the anaerobic induction of fumarate reductase expression.*
- *To test whether the formate induction of the cyx promoter could be mediated by the fhlA gene product, the expression of the cyx-lacZ fusion was examined in an fhlA deletion strain in the presence and in the absence of formate.*

##### SPSS of 0.

Sentences do not even share objects class. A possible case is that sentences share Gene and GC class (the exceptions of SPSS 1 grade) but not the same specific objects; the following pair of sentences is an example of this case:

- *Carbon metabolism regulates expression of the pfl (pyruvate formate-lyase) gene in Escherichia coli.*
- *Later work showed that most mutants lacking either ACDH or ADH activities of the AdhE protein mapped in the adhE gene at 27.9 min [1,4].*

It was clarified that sentences do not necessarily have to contain biological content or refer to RegulonDB’s objects to be annotated and have an SPSS above 0. The annotation assesses the similarity in meaning irrespective of the topic.

Table 3 is a summary of the above-described guidelines.

**Table 3:** Refined rating scale

SPSS	Description
4	Both sentences have in common the same objects and express the same meaning.
3	Both sentences share the same objects and other elements of their meaning. However, one of the sentences lacks relevant elements or refers to the same objects, and it arrives at different conclusions.
2	Both sentences share at least one specific object and some other similarities. In this sense, contrasting conditions are considered related conditions.
1	Both sentences have the same object class in common, but the specific object is different.
0	Sentences do not even share objects of the same class.

#### Annotation process

**Fig. 2:**
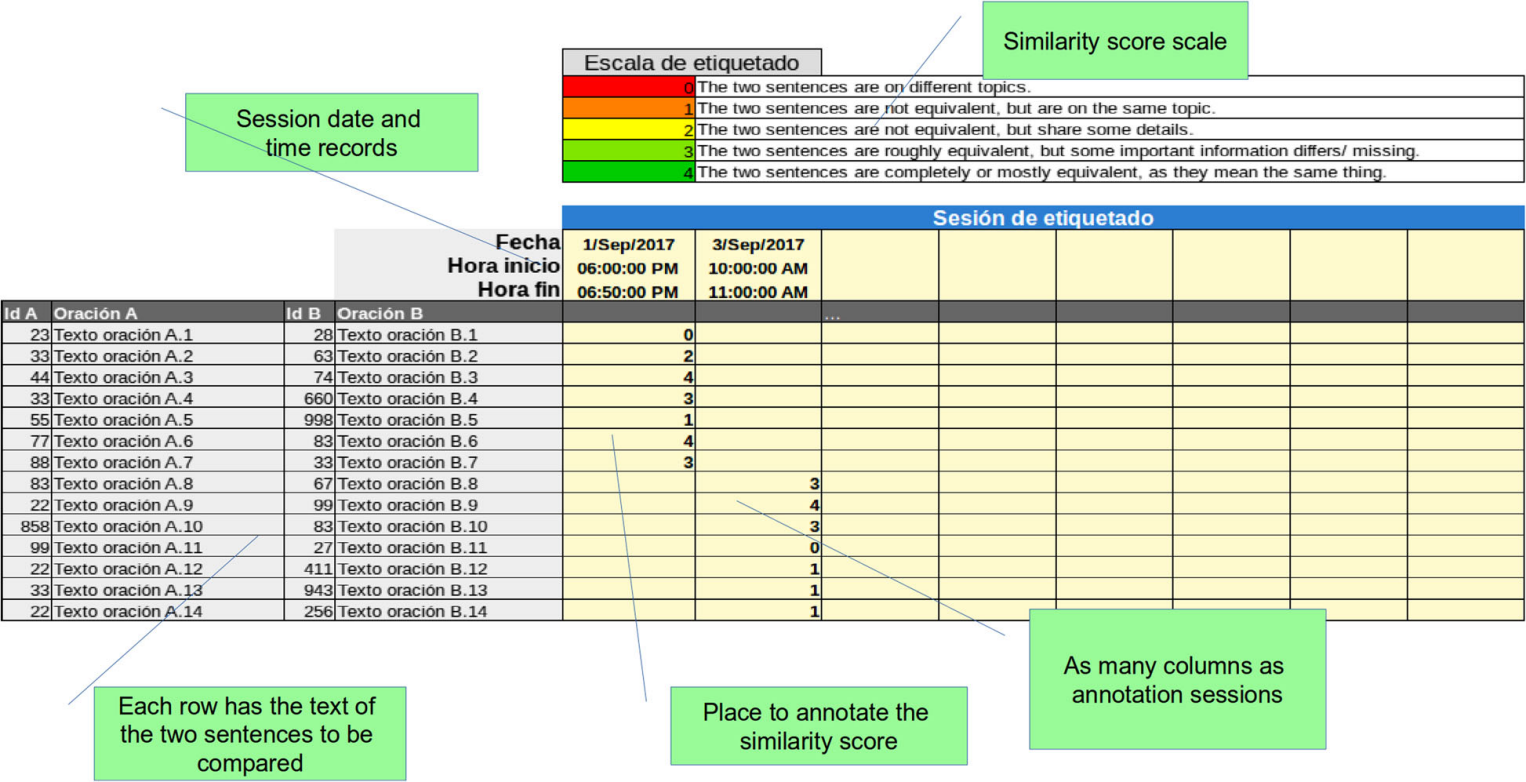
Annotation template. The image shows the spreadsheet template that was used by the annotators. Sentence pairs to be rated are shown in the rows, one sentence pair per row. The cells to the right of each sentence pair were reserved for the annotators’ evaluation, with one annotation session per column. At the top is a rating scale table which was included as a reference

To facilitate the annotation process, we decided to provide annotators with a spreadsheet template (see Fig. 2). The template was designed so that all needed information would be self-contained and the rater did not have to switch to other files. It consisted of a list of all sentence pairs that the annotator had to rate; for each sentence pair, the IDs and text were displayed. The area where the user wrote the scores was organized into columns where each column represented an annotation session, with date and time at the top. A rating scale table was also included as a reference.

The process consisted in: provide each annotator with a file, based on the annotation template, containing exclusively the sentence pairs that have to be evaluated by him/her; annotators had a fixed period of time of one week to rate all pairs; during that period each annotator could divide the rating task into as many sessions as desired as long as he added the session’s date and time; it was indicated that sessions should be exclusive and continuous, i.e., the task should not be interrupted by more than 5 min and annotators should not be performing other tasks in parallel.

The process consisted of providing each annotator with a file, based on the annotation template, containing exclusively the sentence pairs that had to be evaluated by him/her. Annotators had a fixed period of time of 1 week to rate all pairs; during that period, each annotator could divide the rating task into as many sessions as desired, as long as he or she added the session’s date and time. It was indicated that sessions should be exclusive and continuous, i.e., the task should not be interrupted by more than 5 min and annotators should not be performing other tasks in parallel.

It is worth noting that the pairs of sentences assigned to each annotator were randomly selected from the set of pairs.

### Corpus evaluation

The recommended way to evaluate the quality of the resulting corpus is through the Inter-Rater Agreement, also known as Inter-Rater Reliability (IRR) [19, 21–25]. IRR is a measure of the agreement between two or more annotators who have rated an item using a nominal, ordinal, interval, or ratio scale. It is based on the idea that *observed scores* (*O*) are the result of the scores that would be obtained if there were no measurement error—*true scores* (*T*)—plus the *measurement error* (*E*), i.e., *O*=*T*+*E* [21]. One possible source of measurement errors is the measure-instruments instability when multiple annotators are involved. IRR focuses on analyzing how much of the observed scores’ variance corresponds to variance in the true scores by removing the measurement error between annotators. Thus, the reliability coefficient represents how close the given scores (by multiple annotators) are to what would be expected if all annotators had used the same instrument: the higher the coefficient, the better the reliability of the scores.

There are multiple IRR statistics, and which one to use depends on the study design. To select the IRR statistic, some factors should be considered, such as the type of measured variable (nominal, ordinal, etc.), if it is a fully crossed study design or not, and if what it is desired is to measure the annotators’ or the ratings’ reliability.

Our design (see “Annotation design” section) corresponds to a non-fully crossed study design, where an ordinal variable is measured and we are interested in measuring the ratings’ reliability. Having that in mind, the statistics that better accommodated our study were *Fleiss’ Kappa* (Fleiss) [26], *Krippendorff’s Alpha* (Kripp), *Intra Class Correlation* (ICC) [27], *Kendall* (Kendall) [28], and *Gwet’s AC1* (Gwet) [22].

One of the most-used IRR statistics is Cohen’s Kappa analysis (*k*) (5) [29]. It is a relation between *the proportion of units in which the annotators agreed* ($\mathfrak {p}_{o}$) and *the proportion of units for which agreement is expected by chance* ($\mathfrak {p}_{c}$); thus $k = (\mathfrak {p}_{o} - \mathfrak {p}_{c}) / (1 - \mathfrak {p}_{c})$. Originally, this measure was intended for just two annotators who rated all items, so variants were developed in order to fit non-fully crossed study designs with more than two raters per item. The *Fleiss’ Kappa* (1) is a nonweighting measure that considers unordered categories; it was designed for cases when m evaluators are randomly sampled from a larger population of evaluators and each item is rated by a different sample of *m* evaluators. In Eq. (1), *p*_*a*_ represents the averaged extent to which raters agree for the item’s rate and *p*_*ε*_ is the proportion of assignments to the categories.

1 $$ k = \frac{p_{a} - p_{\epsilon}}{1 - p_{\epsilon}}   $$

*Krippendorff’s Alpha* (2) is an IRR measure that is based on computing the disagreement. It provides advantages like being able to handle missing data and handling various sample sizes, and it supports categorical, ordinal, interval, or ratio measured variable metrics. In (2), *D*_*o*_ is the observed disagreement and *D*_*ε*_ is the disagreement one would get if rates were by chance. Thus, it is the ratio between the observed disagreement and the expected disagreement.

2 $$ \alpha = 1 - \frac{D_{o}}{D_{\epsilon}}   $$

*Intra-class correlation* (3) is a consistency measure that can be used to evaluate the ratings’ reliability by comparing the item’s rating variability to the variability of all items and all ratings. It is appropriate for fully crossed as well as for non-fully crossed study designs and when there are two or more evaluators. Another feature is that the disagreement’s magnitude is considered in the computation, as in a weighted Kappa. In (3), *var*(*β*) accounts for variability due to differences in the items, *var*(*α*) is from the variability due to differences in the item’s reevaluations, and *var*(*ε*) is for the variability due to differences in the rating scale used by annotators. Consistent with our study design, we selected the ICC variant as: a “one-way” model, to avoid accounting for systematic deviations among evaluators, because annotators for each item were selected at random. We used the average as the unit of analysis, because all items were rated by an equal number of annotators (i.e., 3).

3 $$ ICC = \frac{var(\beta)}{ var(\alpha) + var(\beta) + var(\epsilon) }   $$

*Kendall’s coefficient* is an association measure that quantifies the degree of agreement among annotators based on the ranking of the items. As a special case of the correlation coefficient, this coefficient will be high when items’ orders (ranked by the given rate) would be similar across annotators. It is based on the computation of the normalized symmetric distances between the ranks. Because it relies on the distances instead of the absolute values, it better handles consistent rater biases, i.e., the bias effect. In (4), *n*_*c*_ refers to the number of concordant and *n*_*d*_ to the number of discordant ranks within a sample of *n* items.

4 $$ W = \frac{ n_{c} - n_{d}}{\frac{1}{2} n(n-1)}   $$

[30] demonstrated that the Kappa coefficient is influenced by trait prevalence (distribution) and base rates, thus limiting comparisons across studies. For that reason, [22] proposed an IRR coefficient (6) that, as Cohen’s Kappa statistic, adjusts the chance agreement—raters agree based on a random rating—to avoid inflating the agreement probability with not true intentional rater’s agreement. However, *Gwet’s coefficient* has the property of not relying on independence between observations; weights are based on weighted dissimilarities. This coefficient presents several advantages: it is less sensitive to marginal homogeneity and positively biases for trait prevalence (more stable); it can be extended to multiple raters; as Krippendorff’s coefficient it can deal with categorical, ordinal, interval, or ratio measures and it can handle missing data; contrary to weighted Kappa, it is not necessary to provide arbitrary weights when applied to ordinal data.

5 $$ Kappa = \frac{p - e(\kappa) }{1 - e(\kappa)}   $$

6 $$ AC = \frac{p - e(\gamma) }{1 - e(\gamma)}   $$

The difference between Gwet and Kappa is in the way that the probability of chance agreement is estimated. In Kappa, *e*(*κ*) is based on combining the estimates of the chance that both raters independently classify a subject into category 1 and estimates the probability of independent classification of a subject into category 2 (7), whereas with Gwet this is based on the chance that any rater (A or B) classifies an item into a category (8).

7 $$ {}e(\kappa) = \left(\frac{A1}{N} \right) \left(\frac{B1}{N} \right) + \left(\frac{A2}{N} \right) \left(\frac{B2}{N} \right)   $$

8 $$ {}\begin{aligned} e(\gamma) &= 2P_{1}(1-P_{1})\\ &= 2 \left(\frac{(A1 + B1)/2}{N} \right) \left(1- \left(\frac{(A1 + B1)/2}{N} \right) \right) \end{aligned}  $$

It is important to note that Gwet proposes 2 variants of its statistic, AC1 and AC2. AC2 is a weighted version—some disagreements between raters are considered more serious than others—of AC1 and thus a better alternative for ordinal data. AC2 is intended to be used with any number of raters and an ordered categorical rating system to rate objects, as is our case. In AC2, both chance agreement as well as misclassification errors are adjusted; thus, it is defined as a “bias-adjusted conditional probability that two randomly chosen raters agree given that there is no agreement by chance” [22].

## Results

### Training period

The training period consisted of 4 iterations in each of which a set of sentence pairs was rated by all annotators. Afterwards, we had a consensus session where conflicts were resolved and questions about the guidelines were answered, resulting in updating the guidelines.

**Fig. 3:**
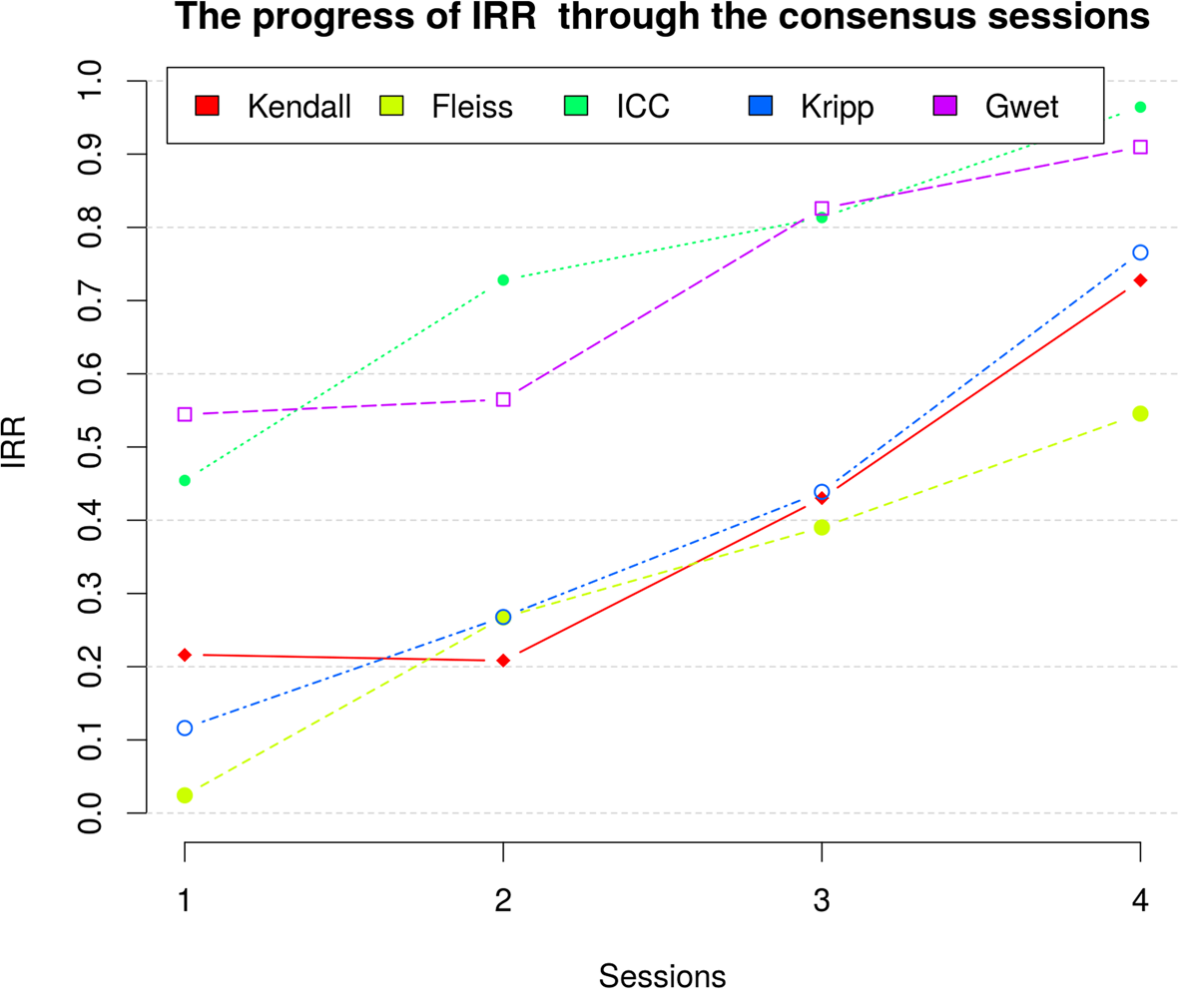
The progress of IRR through the consensus sessions. The chart shows the IRR measured using five different metrics. The IRR score is represented on the y-axis, and the results for the four sessions are chronologically displayed on the x-axis. IRR scores, in all metrics, improved in each subsequent consensus session. For example, the IRR measured using Gwet’s AC2 coefficients improved from 0.545 in the first session to 0.910 in the last one, that is, the annotators’ evaluations were much more homogeneous at the end of the consensus sessions

We performed the IRR analysis of each iteration in order to review the effect of consensus sessions in homogenizing the annotation process. As can be seen in Fig. 3 and Table 4, the grade of interagreement increased in each iteration irrespective of the statistic. In the fourth session, we reached a Fleiss’ Kappa of 0.546 as the lowest metric, which is considered a *moderate* strength of agreement [31]. However, we have to remember that this metric is a nonweighting coefficient, for example, when 2 annotators do not agree on the evaluation of a pair of sentences, for this metric is equally wrong when one annotator grades them with 4 and the other with 0 (i.e., evaluations differ by 4 points) as when one grades them with 2 and the other with 3 (i.e., evaluations differ by only 1 point). That is why we reached an *almost-perfect* IRR in statistics that better deals with ordinal scales: ICC (0.964) and Gwet’s AC2 (0.910). It is noteworthy that Gwet’s coefficients are much more highly recommended methods to compute IRR than those of the Kappa coefficients family.

**Table 4:** IRR through agreement sessions

Session	Kendall	Fleiss	ICC	Kripp	Gwet
1	0.216	0.024	0.454	0.116	0.545
2	0.208	0.267	0.728	0.268	0.565
3	0.430	0.390	0.813	0.439	0.826
4	0.727	0.546	0.964	0.766	0.910

**Fig. 4:**
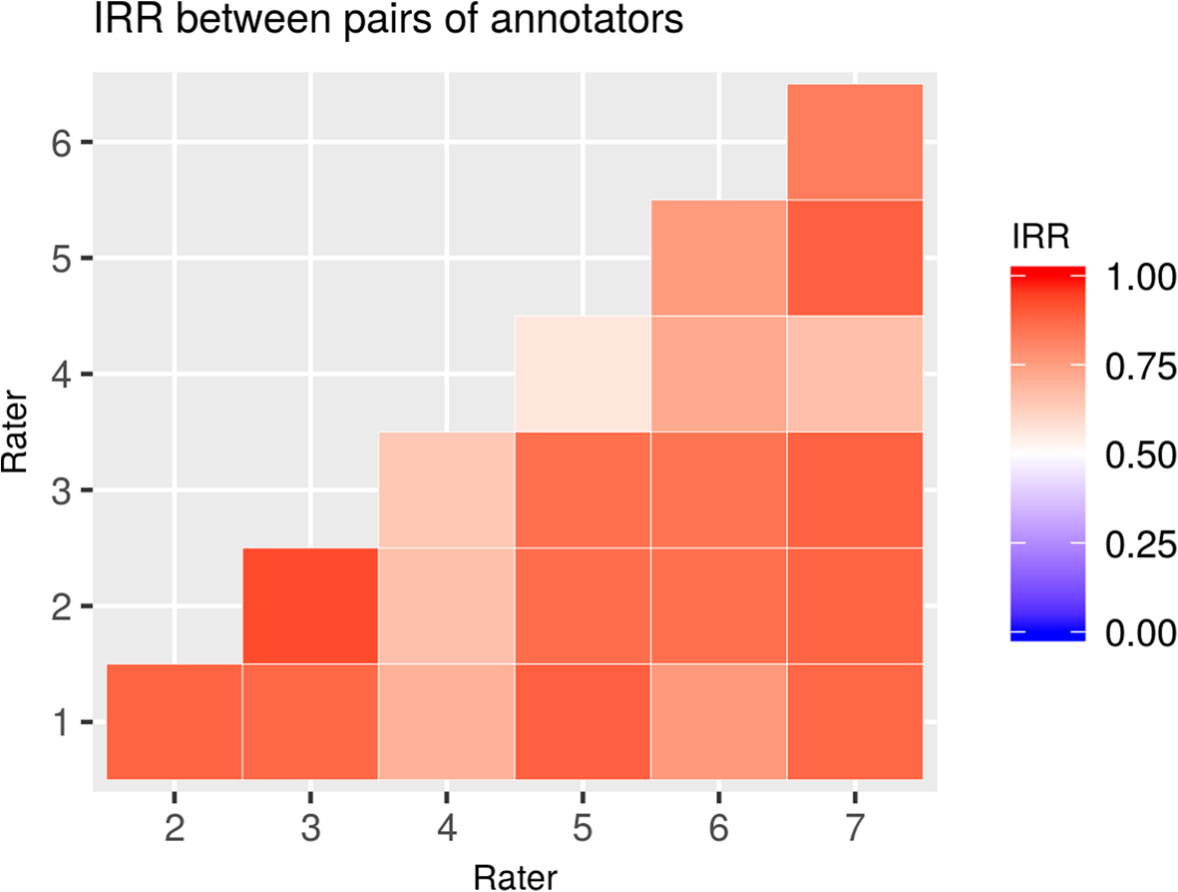
IRR between pairs of annotators at the end of the training sessions. This chart shows the IRR (ICC) of each annotator compared with each of the other annotators. Both x- and y-axes represent annotators; for example, the intersection of the y-value 4 and x-value 5 represents the IRR between annotator-4 and annotator-5. As shown on the IRR scale to the right, the higher the IRR, the more intense the red color, and so in this case, there is a moderate IRR between annotator-4 and annotator-5 and higher agreement between annotators 2 and 3. We noted that annotator-4 had a lower agreement with all others and thus he needed more guideline clarifications

We also compared the IRR between all combinations of annotators’ pairs as a way of detecting consistent bias of one annotator versus the others (see Fig. 4). We determined that more guideline clarifications were needed for annotator 4, who consistently had lower IRR values than the other raters.

### Corpus

After the training period, we built the corpus based on the proposed design (see “Annotation design” section). It resulted in 171 pairs of sentences, each rated by 3 annotators selected by chance from the group of 7 experts. It is noteworthy that the sentences evaluated during the training period were not included in these 171 pairs.

**Fig. 5:**
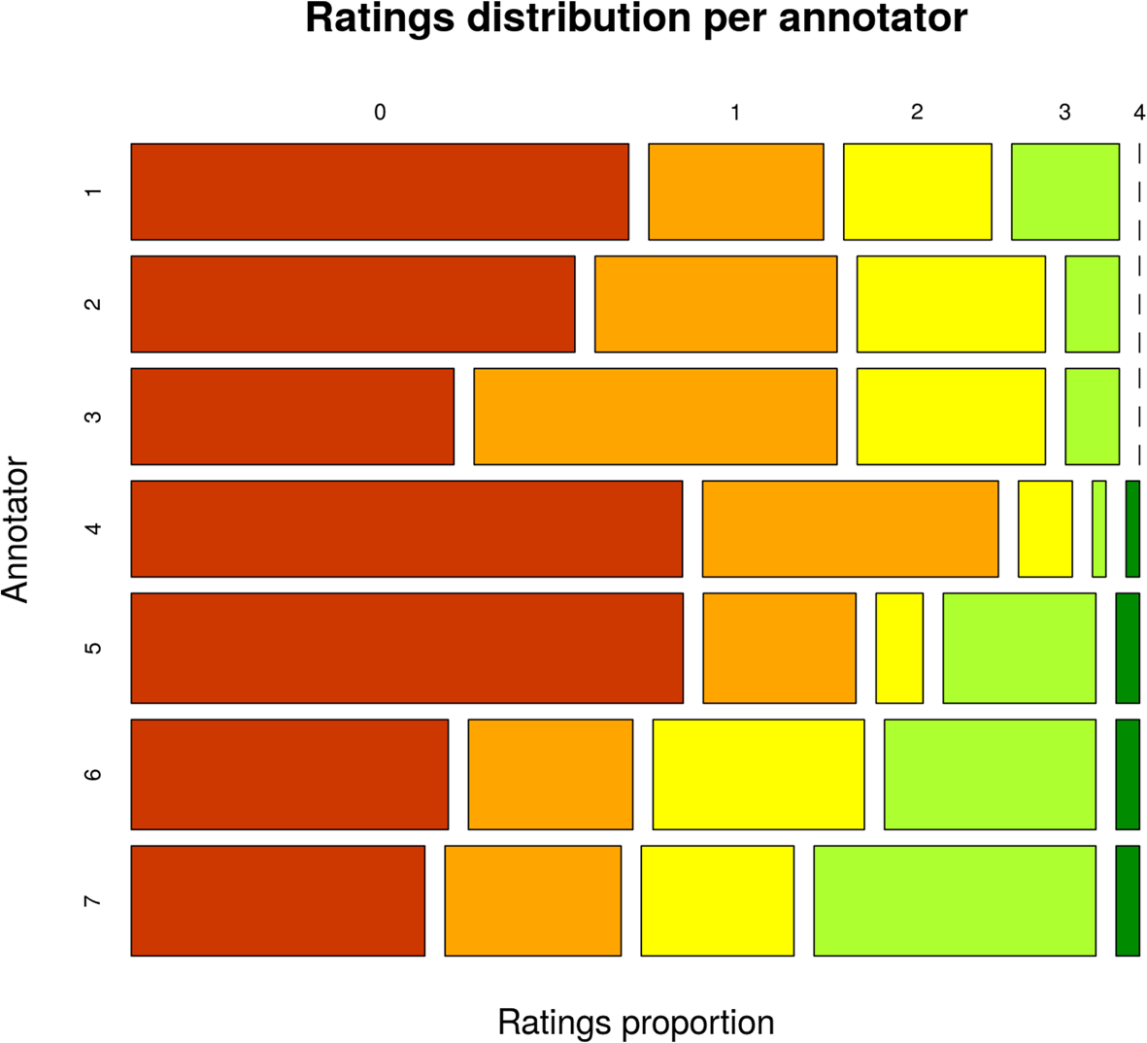
Ratings distribution per annotator. In this chart, the seven annotators are represented on the y-axis, and on the x-axis the evaluation proportions for each similarity grade are represented. Similarity grades are ordered from lowest similarity (0) at the left to highest (4) at the right. For example, it can be seen that both annotator-4 and annotator-5 had the highest proportions of 0-similarity evaluations, but annotator-5 tended to give higher grades in the rest of the cases

**Fig. 6:**
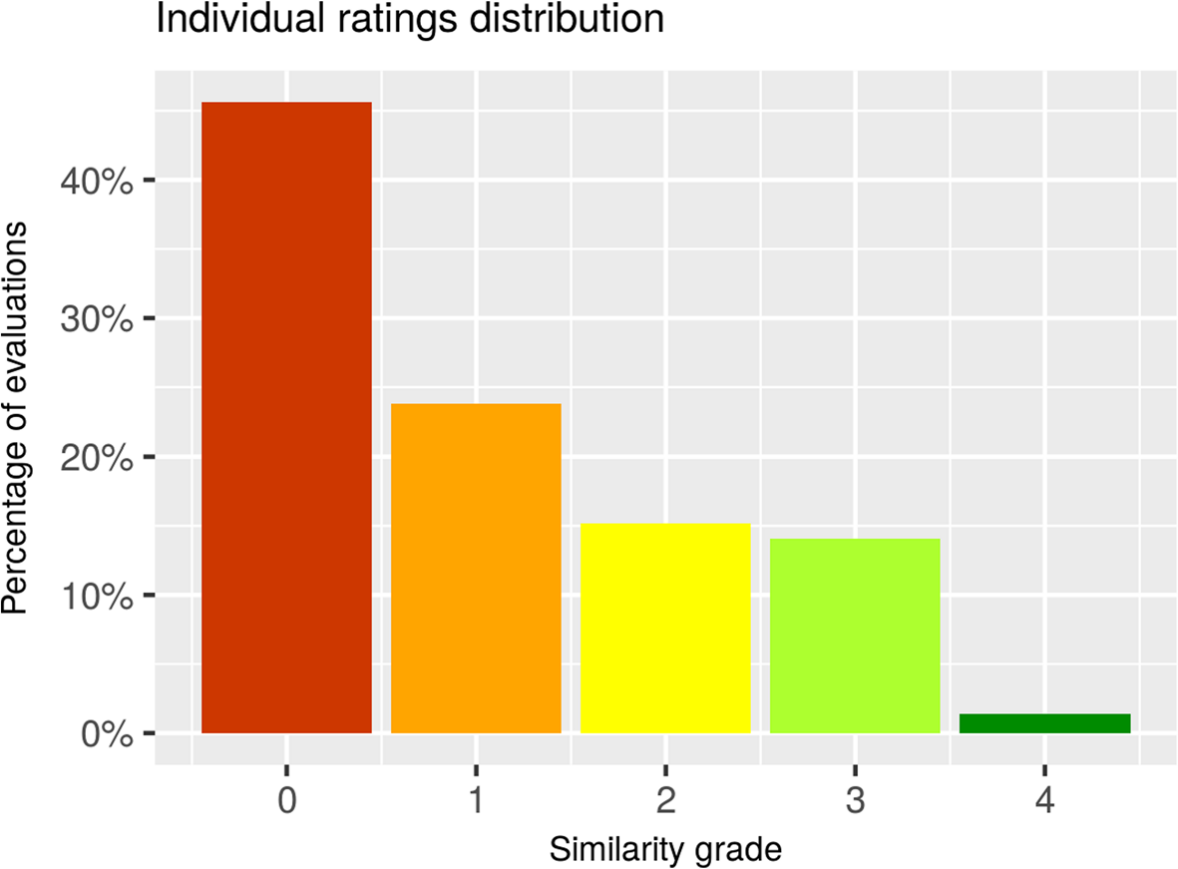
Individual ratings distribution. This chart shows the distribution of annotators’ ratings per similarity grade during the evaluation of the corpus (not the training period). The x-axis shows the five similarity scale values, and the percentage of evaluations within each grade are represented on the y-axis. More than 40% of the evaluations were rated as “no similarities” (score of 0); nevertheless, 50% of evaluations were in the similarity value range between 1 and 3

Several IRR analyses were performed to assess the degree that annotators consistently assigned similarity ratings to sentence pairs (see Table 5). The marginal distributions of similarity ratings did not indicate a considerable bias among annotators (Fig. 5), but they did show a prevalence effect towards lower similarity rates (Fig. 6). A statistic less sensitive to this effect was Gwet’s AC, which makes an appropriate index of IRR, in particular the AC2 variant, due to the ordinal nature of our data. The resulting coefficient indicated *very good agreement* [32] of *AC*2=0.8696 with a 95% confidence interval [0.8399, 0.8993].

**Table 5:** Corpus’ inter-rate agreement for various statistics

Statistic	Variable	Value	*p*-value
Fleiss’ Kappa	Kappa	0.443	0
Krippendorff’s Alpha	Alpha	0.745	
Kendall’s coefficient	W	0.741	7.86e-18
Intraclass Correlation	ICC	0.919	6.7e-83
Gwet’s coefficient	AC2	0.870	0

For the sake of clarity, we investigated if the non-fully crossed design caused too-inflated coefficients. To do this, we first grouped the sentence pairs by the annotators who rated them (now each of these groups could be considered a fully crossed study design); next, we computed the IRR for each group; finally, we computed the arithmetic mean of all groups. The resulting averages (Table 6) were quite similar to coefficients computed for the whole corpus, reconfirming the corpus reliability.

**Table 6:** IRR by annotators group

Annotators	Kendall	Fleiss	ICC	Kripp	Gwet
1, 2, 3	0.782	0.597	0.941	0.814	0.864
1, 2, 7	0.641	0.512	0.926	0.705	0.894
1, 6, 7	0.788	0.358	0.912	0.756	0.686
2, 3, 4	0.669	0.442	0.916	0.691	0.907
3, 4, 5	0.712	0.310	0.894	0.708	0.802
4, 5, 6	0.593	0.268	0.753	0.602	0.818
5, 6, 7	0.833	0.409	0.913	0.772	0.784
Mean	0.717	0.414	0.894	0.721	0.822

From the individual rating distribution (Fig. 6), we can see that although the distribution is biased towards no similarity, we achieved a good amount (> 50%) of sentence pairs rated within the 1-3 score range.

## Discussion

We observed that the IRR increased more significantly after the third training session. We think that this increase can be explained mainly by two factors. First, annotators familiarized themselves with the guidelines and they had a better understanding of what was expected for the task. Despite task explanations and annotation guidelines, in the first sessions there was a tendency to grade the similarity of the biological objects mentioned in the compared texts and to overlook the full semantics conveyed by those texts. Second, after the first two sessions, annotators had collected a good set of examples along with the respective explanatory notes from the previous consensus sessions. These examples served as disambiguation sources when needed. It is interesting that both factors are related to the hypothesis that although similarity is an intuitive process, there is not a perfect consensus, especially about the grades of similarity [33–36]. It depends on the personal context, and we could confirm the importance of guidelines and consensus sessions to homogenize, at a certain grade, the annotators performance.

Another practice that we found helpful during the consensus sessions was the participation of a mediator who was familiarized with the guidelines and with the task’s goal but was not part of the annotators group, i.e., a third party. When needed, the mediator’s role was limited to exhort annotators to explain their posture and, if pertinent and possible, to put the discussion in equivalent terms through a general context analogy. This helped to avoid unjustified influence of those annotators who were more experienced or who upheld more strongly their opinions.

In general, annotators agreed that the sentences without biological objects mentions were more difficult to assess and that in the candidate sentences there was a clear bias toward low similarity scores. This similarity dataset is just the first iteration of an ongoing process. We plan to repeat this strategy to extend the dataset; instead of using the basic STS process, now we could use a similarity model trained with the current corpus [18], and therefore it is reasonable to expect an improvement in the preselection step, more likely resulting in a more balanced rating distribution, i.e., more grades of 3 and 4.

In the spirit of weighing the size and distribution of our corpus against previous work, we compared it with BIOSSES. We selected this corpus because, to the best of our knowledge, it is the only similarity corpus specialized for the biomedical domain, and setting our corpus side by side with the general-domain ones (e.g., MSRP, SEMEVAL, ULPC) would be unfair. Regarding balance for these two corpora with respect to the number of sentence pairs per grade, BIOSSES is better balanced, with 15% of sentences graded with a value of 0, 12% with 1, 27% with 2, 35% with 3, and 11% graded with 4. Our corpus has a distribution of 48%, 22%, 15%, 14%, and 1% corresponding to the 0, 1, 2, 3, and 4 similarity grades. However, concerning corpora size, although it is still small our corpus, with 171 sentence pairs, is 70% larger than the BIOSSES corpus, which consists of only 100 pairs of sentences. Moreover, even though BIOSSES is specialized for the biomedical domain, its coverage is still too broad for our purpose. This is evidenced by the fact that, when analyzing terms’ frequencies in BIOSSES, within the top 50 terms we found terms like cell, tumor, cancer, study, report, human, gene, lung, leukemia, etc., whereas in our corpus the prevailing terms are site, expression, activation, gene, protein, strain, regulation, DNA, region, downstream, upstream, etc.

We believe that our publicly available dataset (see “Availability of data and materials” section) can be of great benefit in several NLP applications. For example, we are already successfully using it to fine-tune and test a semantic similarity engine as part of an assisted curation pipeline. Within these experiments, we used an ensemble of similarity metrics that were string, distributional, and ontology based. The individual measures were combined through different regression models which were trained using the corpus presented in this publication. Our models obtained strong correlations (*ρ*=0.700) with human evaluations, which are far from state-of-the-art in general domains but are quite good considering our highly specialized domain—Microbial Transcriptional Regulation. In the absence of this corpus, the only alternative would have been to equally weight the different metrics, which in our experiments results in a Pearson’s correlation (*ρ*) of 0.342, at best. With these experiments, it was shown that this corpus is not only relevant but also useful for applied tasks [18].

## Conclusions

We did not obtain a corpus with ratings as balanced as desired; however, we now have a good representation of 4 of the 5 rates and a corpus with very good IRR. Therefore, it is going to serve well our purposes, and we think it can be quite a valuable starting point, with respect to data and processes to continue building a standard similarity corpus in the transcriptional regulation literature. To the best of our understanding, this is the first similarity corpus in this field, and thus it represents a stepping stone towards the evaluation and training of NLP-based high-throughput curation of literature on microbial transcriptional regulation.

## Abbreviations

DNA: Deoxyribonucleic acid
FNR: Fumarate and nitrate reductase regulatory protein
Fleiss: Fleiss’Kappa coefficient
GC: Growth conditions
Gwet: Gwet’s AC1 statistic
ICC: Intraclass correlation
IRR: Interrater reliability
Kendall: Kendall coefficient
Kripp: Krippendorff’s Alpha coefficient
MSRP: Microsoft Research Paraphrase
NLP: Natural Language Processing
RI: Regulatory interaction
RNA: Ribonucleic acid
SEMEVAL: Semantic Evaluation workshop
SPSS: Sentence Pair Similarity Score
STS: Semantic Textual Similarity
TF: Transcription Factor(s)
TFBS: TF binding site
TU: Transcription Unit
ULPC: User Language Paraphrase Corpus

## Footnotes

1
http://regulondb.ccg.unam.mx/

2
http://regulondb.ccg.unam.mx/menu/tools/nlp/index.jsp

3 Declarative, interrogative, exclamatory, etc.

4 Based on the stylographic tag assigned by our home-made PDF processing tool.

5 Applying a baseline metric.

6 In *fully crossed design studies* all evaluated items (pairs of sentences) are rated by the same set of annotators, whereas in *non-fully crossed design studies*, different items are rated by different subsets of annotators.
